# Real-world treatment patterns and patient-reported outcomes in episodic and chronic migraine in Japan: analysis of data from the Adelphi migraine disease specific programme

**DOI:** 10.1186/s10194-019-1012-1

**Published:** 2019-06-07

**Authors:** Kaname Ueda, Wenyu Ye, Louise Lombard, Atsushi Kuga, Yongin Kim, Sarah Cotton, James Jackson, Tamas Treuer

**Affiliations:** 1 Eli Lilly Japan K.K., 5-1-28, Isogamidori, chuo-ku, Kobe-shi, 651-0086 Japan; 2 Eli Lilly and Company, Lilly Corporate Center, Indianapolis, IN 46285 USA; 3 Adelphi Mill, Grimshaw Lane, Bollington, Cheshire, SK10 5JB UK; 4 Lilly Hungária Kft, Madách Imre út 14, Budapest, 1075 Hungary

**Keywords:** Chronic migraine, Episodic migraine, Migraine, Clinical practice, Treatment patterns, Patient-reported outcomes

## Abstract

**Background:**

In Japan, detailed information on the characteristics, disease burden, and treatment patterns of people living with migraine is limited. The aim of this study was to compare clinical characteristics, disease burden, and treatment patterns in people with episodic migraine (EM) or chronic migraine (CM) using real-world data from clinical practice in Japan.

**Methods:**

This was an analysis of data collected in 2014 by the Adelphi Migraine Disease Specific Programme, a cross-sectional survey of physicians and their consulting adult patients in Japan, using physician and patient questionnaires. We report patient demographics, prescribed treatment, work productivity, and quality-of-life data for people with CM (≥15 headache days/month) or EM (not fulfilling CM criteria). In descriptive analyses, continuous and categorical measures were assessed using t-tests and Chi-squared tests, respectively.

**Results:**

Physicians provided data for 977 patients (mean age 44.5 years; 77.2% female; 94.5% with EM, 5.5% with CM). A total of 634/977 (64.9%) invited patients (600 with EM; 34 with CM) also provided data. Acute therapy was currently being prescribed in 93.7% and 100% of patients with EM and CM, respectively (*p* = 0.069); corresponding percentages for current preventive therapy prescriptions were 40.5% and 68.5% (*p* < 0.001). According to physicians who provided data, preventive therapy was used at least once by significantly fewer patients with EM than with CM (42.3% vs. 68.5%, respectively; *p* < 0.001). Among patients who provided physicians with information on issues with their current therapy (acute therapy: *n* = 668 with EM, *n* = 38 with CM; preventive therapy: *n* = 295 with EM, *n* = 21 with CM), lack of efficacy was the most frequently identified problem (acute therapy: EM 35.3%, CM 39.5% [*p* = 0.833]; preventive therapy: EM 35.3%, CM 52.4% [*p* = 0.131]). Moderate-to-severe headache-related disability (Migraine Disability Assessment total score ≥ 11) was reported by significantly fewer patients with EM than with CM (21.0% vs. 60.0%, respectively; *p* < 0.001) among patients who provided data.

**Conclusions:**

Preventive treatment patterns in people with EM versus CM differ in Japan, with both types of migraine posing notable disease burdens. Our findings demonstrate that more effective migraine therapies are required to reduce the burden of the disease.

**Electronic supplementary material:**

The online version of this article (10.1186/s10194-019-1012-1) contains supplementary material, which is available to authorized users.

## Background

Migraine is a debilitating primary headache disorder, characterized by recurrent unilateral (one-sided) pulsatile headaches. According to the Headache Classification Committee of the International Headache Society (IHS), people with ≥15 headache days per month (HDM) for more than 3 months, at least 8 of which meet criteria for a migraine attack and/or respond to acute migraine-specific medication, are classified as having chronic migraine (CM) [1]; those with ≤14 HDM are classified as having episodic migraine (EM) [2].

The impact of migraine extends far beyond the physical pain of a migraine attack, with substantial effects on multiple aspects of an individual’s life, including health-related quality of life (HRQoL) and day-to-day functioning [3, 4]. In 2016, the Global Burden of Diseases, Injuries, and Risk Factors study found migraine to be the second leading cause of years lived with disability (YLD) (after lower back pain), contributing 45.1 million of total YLDs globally [5].

Recent data indicate that migraine is highly prevalent worldwide: a systematic review and meta-analysis published in 2017 estimated the average global prevalence of migraine at the community level to be 11.6% (10.4% in Africa, 10.1% in Asia, 11.4% in Europe, 9.7% in North America, and 16.4% in Central and South America) [6].

The overall prevalence of migraine in Japan was reported to be 8.4% (6.6% in men, 13.0% in women) in 1997 [7]). In 2004, the Daisen study, a population-based survey conducted in the Daisen area of the Tottori prefecture, reported a prevalence for migraine of 6.0% in residents aged 20 years or older. Migraine was more prevalent in women than in men (9.1% vs. 2.3%, respectively), with the highest rates among women aged 30–49 years [8]. Other surveys, conducted in Japanese high-school students, reported prevalences for migraine of 5.8% (5.5% in boys, 6.1% in girls) in 2005 [9] and 4.8% (3.3% in boys, 6.5% in girls) in 2007 [10].

Overall, most of the participants in these epidemiological surveys had not consulted medical facilities, despite migraine being the cause of notable disability in their everyday lives [8–10].

The *Clinical Practice Guideline for Chronic Headache* [11], published in 2013 by the Japanese Society of Neurology (JSN) and the Japanese Headache Society (JHS), serves as a helpful guide for healthcare professionals who treat people with migraine in Japan. However, only limited information is available on the characteristics, disease burden, and treatment patterns of people with EM or CM in Japan, in contrast to that reported for some other countries (e.g., the USA). The aim of the current study was to provide this information, using real-world data reported by people with EM or CM actively seeking treatment for their migraine in Japan and by their treating physicians, focusing on clinical characteristics (including comorbidities), acute and preventive treatments, concomitant medications, work productivity, and HRQoL. Additionally, the study sought to examine the burden of illness and disability across patient subgroups based on average monthly headache frequency.

## Methods

### Study design and population

This research was a retrospective analysis of cross-sectional survey data collected by the Japanese Adelphi Migraine Disease-Specific Programme (DSP) in 2014, which was conducted independently by Adelphi Real World (Bollington, UK). The Japanese Adelphi Migraine DSP surveyed physicians and their consulting adult patients with migraine and collected migraine-specific data pertaining to treatment practice, symptom prevalence, patient demographics, clinical outcomes, medication utilization, adherence patterns, healthcare utilization, work productivity, and HRQoL. The survey utilized the general methodology for Adelphi Migraine DSP surveys [12].

The study population comprised physicians (internists and neurologists) treating people with migraine and their migraine-diagnosed patients who were actively seeking care from their healthcare provider. The recruitment of physician and patient participants is described in Ford et al. [13]. Participating physicians were required to recruit ten patients who had a diagnosis of migraine. The first nine patients were to be consecutive, but to achieve a 10% oversampling of patients who had failed at least one prior preventive treatment, the tenth patient had to meet this requirement and may not have been consecutive.

Physicians were asked to complete a patient record form (PRF) for all patients (data from PRFs are referred to as physician-reported data throughout). The PRF asked detailed questions on patient demographics, diagnoses, severity of migraine, number of headache days, comorbidities, migraine diagnosis, monitoring, treatment history (acute and preventive), drivers of migraine therapy choice, adherence to therapy, and general patient management (including the frequency of consultations with the treating and other physicians).

Once they had completed each PRF, the physician then invited the patient to complete a confidential patient self-completion (PSC) form (data from PSC forms are referred to as patient-reported data throughout). The PSC form was designed to elicit greater detail on the patient’s demographics, level of satisfaction with their migraine treatment, adherence to therapy, and health insurance status. The PSC form also assessed validated patient-reported outcome measures, including the Migraine Disability Assessment (MIDAS) [14, 15], the EuroQol-5 Dimensions (EQ-5D) questionnaire [16], and the Work Productivity and Activity Impairment (WPAI) questionnaire [17]. The MIDAS was designed to quantify headache-related disability over a 3-month period [14, 15]. The EQ-5D is a generic, multidimensional, HRQoL instrument that uses a health status profile and a visual analog scale (VAS) to rate global HRQoL [16]. The WPAI is a valid and reliable instrument for measuring work productivity, which captures missed work time, general health perceptions, physical and emotional role, pain, symptom severity, and work and usual activity impairment [17]. The WPAI in this study specifically captured the implications for work productivity and usual activity impairment due to headaches. Completion of the PSC form was at the discretion of the patient.

### Statistical methods

Data were collected cross-sectionally (January to March 2014). After deidentification, data were quality checked, and final data were entered into an electronic database. These analyses used all available data from the 2014 Japan Adelphi Migraine DSP cross-sectional database for all patients who met the stated requirements for this study. On the basis of physician-reported data, patients were classified into two groups: EM (not fulfilling CM criteria) or CM (≥15 HDM), in line with a previous Adelphi Migraine DSP survey [13]. Additionally, four subgroups were identified (EM 0–3, 4–7, and 8–14 HDM, and CM ≥15 HDM) according to HDM frequency.

Descriptive statistical analyses were performed to compare clinical characteristics, disease burden, and treatment patterns between patients with EM and CM, and among the four subgroups. Two sample t-tests were used for continuous measures, and Fisher’s exact (for small sample sizes) or Chi-squared tests were used for categorical measures when comparing patients with EM and CM.

Analysis of variance (ANOVA) was used to examine differences in mean disease disability, HRQoL, and work productivity scores across the four subgroups.

Summary statistical information was based on non-missing data. Statistical test results were conducted at a two-sided 5% significance level. No adjustments were made for multiple comparisons. Analyses were conducted using SAS Enterprise Guide 7.12 (SAS Institute, Cary, NC, USA).

### Results

A total of 114 physicians (64 internists, 50 neurologists) provided PRFs for 1019 patients with migraine, of which 977 were for adults and contained information on HDM. Of 977 invited patients, 634 (64.9%; 600 with EM, 34 with CM) also provided relevant data in PSC forms.

### Patient characteristics

According to physician-reported data from 977 PRFs, the mean age of patients with migraine was 44.5 years; most had EM (94.5%) and were female (77.2%). The average number of migraine HDM was reported as 3.0 in patients with EM and 10.8 in those with CM (*p* < 0.001). The characteristics of patients with EM and CM were comparable (*p* > 0.05), with the exceptions that tension-type headache and medication-overuse headache (MOH) were significantly more common in patients with CM than in those with EM (*p* ≤ 0.002) (Table 1). Half (50.2%) of patients with EM had 0–3 HDM, 29.2% had 4–7 HDM, and 15.1% had 8–14 HDM.

**Table 1:** Characteristics of patients with episodic migraine and chronic migraine: physician-reported data

Patient characteristic	Cohort		Total (*N* = 977)	*p*-value
	Episodic migraine (*N* = 923)	Chronic migraine (*N* = 54)		
Age (years), mean (SD)	44.4 (14.6)	46.3 (13.2)	44.5 (14.5)	0.351^a^
Female, n (%)	711 (77.0)	43 (79.6)	754 (77.2)	0.658^b^
Body mass index (kg/m^2^), mean (SD)	21.7 (3.3)	21.9 (3.3)	21.7 (3.3)	0.627^a^
Smoking status, n (%)				0.200^b^
Current smoker	105 (11.8)	9 (17.3)	114 (12.1)	
Ex-smoker	90 (10.1)	2 (3.8)	92 (9.7)	
Has never smoked	698 (78.2)	41 (78.8)	739 (78.2)	
No. of migraine headache days (average per month), mean (SD)	3.0 (2.8)	10.8 (7.8)	3.4 (3.8)	< 0.001^a^
Also had tension-type headaches, n (%)	194 (21.0)	21 (38.9)	215 (22.0)	0.002^b^
Also had medication-overuse headaches, n (%)	36 (3.9)	14 (25.9)	50 (5.1)	< 0.001^c^
Home circumstances, n (%)				
Lives with spouse/partner	515 (57.7)	34 (66.7)	549 (58.2)	0.205^b^
Lives with other family	204 (22.8)	15 (29.4)	219 (23.2)	0.280^b^
Lives with friends	1 (0.1)	0	1 (0.1)	1.000^c^
Lives alone	190 (21.3)	5 (9.8)	195 (20.7)	0.049^b^
Employment status, n (%)				0.131^c^
Full-time	433 (48.0)	23 (45.1)	456 (47.8)	
Part-time	126 (14.0)	3 (5.9)	129 (13.5)	
Student	35 (3.9)	1 (2.0)	36 (3.8)	
Unemployed	104 (11.5)	8 (15.7)	112 (11.8)	
Retired	12 (1.3)	3 (5.9)	15 (1.6)	
Homemaker	187 (20.7)	13 (25.5)	200 (21.0)	
Other	5 (0.6)	0	5 (0.5)	
Patient with any comorbidity, n (%)	455 (49.3)	29 (53.7)	484 (49.5)	0.529^b^
Total no. of comorbidities (max = 37)^d^, mean (SD)	1.8 (1.2)	2.1 (1.8)	1.8 (1.3)	0.378^a^

Continuous variables are reported as mean (standard deviation [SD]) for non-missing observations; percentages are calculated as proportion of non-missing data

^a^t-test

^b^Chi-squared test

^c^Fisher’s exact test

^d^Mean number of comorbidities among patients with comorbidities

The most frequently physician-reported migraine-related symptoms considered most troublesome to patients (occurring in ≥5% of patients with EM or CM) were pain related: unilateral pain (50.8% of patients), pulsating/throbbing pain (20.8%), bilateral pain (17.7%), and pain worsened by activity (8.5%) in patients with EM (*n* = 896), and unilateral pain (37.7%), pulsating/throbbing pain (37.7%), bilateral pain (24.5%), pain worsened by activity (7.5%), nausea (7.5%), and vomiting (7.5%) in those with CM (*n* = 53). Pulsating/throbbing pain was significantly (*p* = 0.004) more of a problem for those with CM than those with EM. Photophobia was reported by 1.5% and 3.8%, and phonophobia was reported by 0.9% and 0%, of patients with EM and CM, respectively.

Approximately half of all patients with EM (*n* = 455 [49.3%]) or CM (*n* = 29 [53.7%]) were reported by physicians as having comorbidities (Table 1). The most common comorbid conditions seen in these patients in both migraine groups (≥5% of those with EM or CM experiencing comorbidities) were hypertension (27.5% and 27.6%, respectively), hyperlipidemia (19.3% and 20.7%), anxiety (18.0% and 13.8%), sleep disorders (12.7% and 17.2%), gastrointestinal disorders/dyspepsia (13.6% and 24.1%), depression (11.2% and 17.2%), obesity (8.8% and 13.8%), respiratory problems (asthma/chronic obstructive pulmonary disease/allergic rhinitis; 12.5% and 6.9%), diabetes (6.4% and 6.9%), panic disorder (6.4% and 3.4%), and menstrual disorders (3.1% and 6.9%) (Additional file 2: Figure S1). There was no significant difference in the incidence of any comorbidity between patients with CM and EM. Concomitant medications were as expected for these comorbidities and are detailed in Additional file 1: Table S1.

### Treatment

According to physician-reported data, over 85% of patients were consulting the physician about migraine during the visit in which they completed their PSC form (Table 2). Most patients with EM were consulting an internist (58.2%), whereas a majority of those with CM were seeing a neurologist (74.1%; Table 2). However, a greater proportion of patients with CM who were not currently receiving preventive therapy were consulting an internist (9/17 [52.9%]) compared with 26% of the overall CM cohort. During the previous 12 months, similar mean numbers of internist/neurologist consultations for migraine were made by patients with EM (6.3 and 6.1, respectively) and CM (4.6 and 6.5, respectively), and migraine-related hospital visits were recorded for similar percentages of each group (28.2% and 31.5%, respectively) (Table 2).

**Table 2:** Physician consultations and healthcare resource utilization: physician-reported data

Healthcare resource	Cohort		Total (*n* = 977)	*p*-value
	Episodic migraine (*n* = 923)	Chronic migraine (*n* = 54)		
Doctor specialty, n (%)				< 0.001^a^
Internist	537 (58.2)	14 (25.9)	551 (56.4)	
Neurologist	386 (41.8)	40 (74.1)	426 (43.6)	
Current consultation was for migraine, n (%)	794 (86.1)	51 (94.4)	845 (86.6)	0.081^a^
No. of times patient consulted for migraine in the past 12 months – internist, mean (SD)	6.3 (5.4)	4.6 (4.9)	6.2 (5.4)	0.193^b^
No. of times patient consulted for migraine in the past 12 months – neurologist, mean (SD)	6.1 (4.7)	6.5 (5.7)	6.1 (4.8)	0.576^b^
No. of patients making hospital visits for migraine in the past 12 months, n (%)	260 (28.2)	17 (31.5)	277 (28.4)	0.603^a^
Ever had surgical intervention for migraine condition, n (%)	1 (0.1)	0	1 (0.1)	1.000^c^

Continuous variables are reported as mean (standard deviation [SD]) for non-missing observations; percentages are calculated as proportion of non-missing data

^a^Chi-squared test

^b^t-test

^c^Fisher’s exact test

**Fig. 1:**
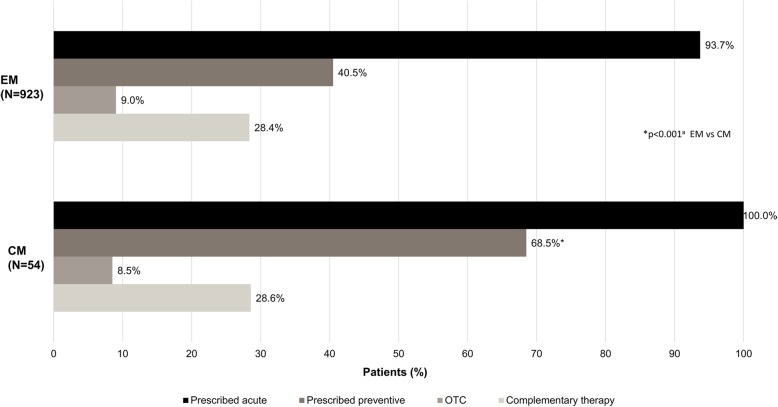
Acute and prophylactic treatment of patients with episodic migraine (EM) and chronic migraine (CM): physician-reported data. Percentages are calculated as proportion of non-missing data. OTC, over the counter. ^a^Chi-squared test

Most patients with EM (93.7%) or CM (100%) were currently being prescribed an acute treatment for their migraine; 40.5% of those with EM and 68.5% of those with CM had current prescriptions for preventive therapy (*p* < 0.001) (Fig. 1). One-third of patients with EM (34.2%) and two-thirds (68.5%) of those with CM were being prescribed both acute and preventive therapy. Only 9.0% and 8.5% of patients with EM or CM, respectively, were taking over-the-counter medications (mostly nonsteroidal anti-inflammatory drugs [NSAIDs] and acetaminophen/ethenzamide); the use of complementary therapies (most commonly massage, relaxation therapy, and Pilates/yoga) was 28.4% and 28.6% in patients with EM and CM, respectively.

In both patients with EM and those with CM who were receiving therapy, the majority of acute treatment prescriptions were for triptans and NSAIDs (Table 3). Current use of opioids as acute therapy was low (0.2% of patients with EM; no patients with CM). Preventive therapy prescriptions most frequently comprised anticonvulsants and calcium antagonists (Table 3). Notable proportions of patients with EM or CM who were receiving preventive therapy were being prescribed the antidepressant/anxiolytic, amitriptyline, as the preventive therapy. Among patients with a current prescription for preventive therapy, the use of lomerizine, valproic acid, and amitriptyline was significantly higher in those with CM than in those with EM.

**Table 3:** Medications currently prescribed as acute or preventive migraine treatment for ≥5% of patients with episodic and chronic migraine: physician-reported data

Medication	Cohort		Total (*n* = 977)	*p*-value
	Episodic migraine (*n* = 923)	Chronic migraine (*n* = 54)		
Acute treatment				
Eletriptan	91 (9.9)	2 (3.7)	93 (9.5)	0.134^a^
Naratriptan	133 (14.4)	11 (20.4)	144 (14.7)	0.230^a^
Sumatriptan	162 (17.6)	7 (13.0)	169 (17.3)	0.386^a^
Rizatriptan	148 (16.0)	14 (25.9)	162 (16.6)	0.057^a^
Zolmitriptan	113 (12.2)	6 (11.1)	119 (12.2)	0.805^a^
Loxoprofen	275 (29.8)	18 (33.3)	293 (30.0)	0.581^a^
Diclofenac	48 (5.2)	4 (7.4)	52 (5.3)	0.525^b^
Preventive treatment				
Lomerizine	131 (14.2)	13 (24.1)	144 (14.7)	0.046^a^
Valproic acid	116 (12.6)	17 (31.5)	133 (13.6)	< 0.001^a^
Amitriptyline	61 (6.6)	8 (14.8)	69 (7.1)	0.048^b^

Data are n (%)

^a^Chi-squared test

^b^Fisher’s exact test

According to physician-reported data, acute therapy was used at least once (as first or higher line of therapy) by 95.2% and 100.0% of patients with EM or CM (*p* = 0.167), and preventive therapy was used at least once by 42.3% and 68.5% of patients with EM or CM (*p* < 0.001) (Additional file 3: Figure S2).

**Fig. 2:**
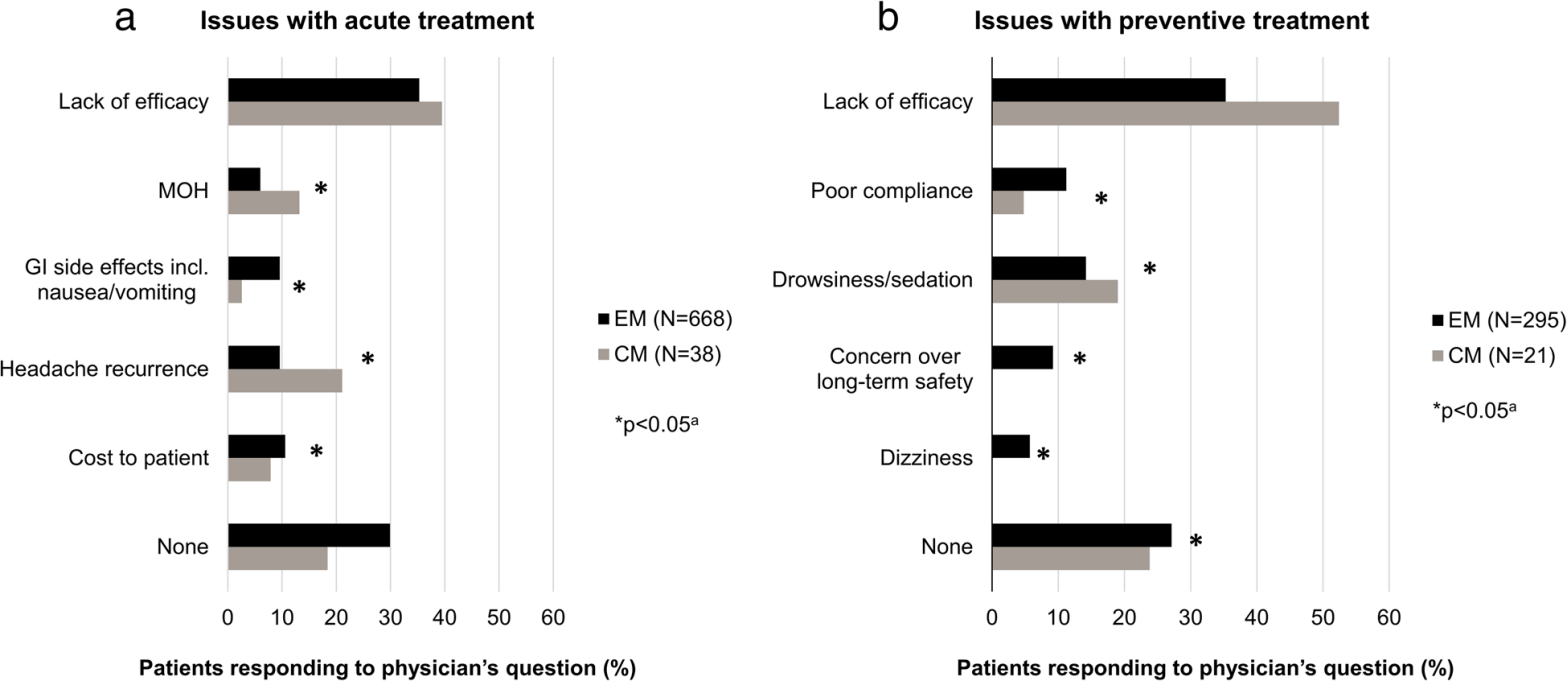
Issues with (**a**) acute and (**b**) prophylactic treatment reported for > 5% of patients with episodic migraine (EM) and chronic migraine (CM): physician-reported data. Issues were selected from a predetermined list by physicians when patients responded to the question “Any issues with acute/preventive regimen?”. An item was checked only if considered an issue by the physician. Data were not available for all patients (either unknown or patient was not receiving treatment). Percentages are calculated as proportion of patients receiving acute therapy who reported at least one issue with therapy (acute treatment: EM, *N* = 668; CM, *N* = 38, total, *N* = 706; preventive treatment: EM, *N* = 295; CM, *N* = 21; total, *N* = 316). “None” indicates the physician noted the patient had no acute/preventive treatment issues in the “other” category. GI, gastrointestinal; MOH, medication overuse headache. ^a^Fisher’s exact test

Physicians reported that 51–60% and 23–30% (percentages based on total patient cohorts, although data were not available for all patients [either unknown or patients were not receiving therapy]) of patients with EM or CM indicated they were experiencing at least one issue with their current acute or preventive therapy, respectively (selected from a predetermined list of items in response to the question “Any issues with acute/preventive regimen?”). Among patients for whom PRF data on issues with acute therapy were available (668 with EM; 38 with CM), lack of efficacy was the most frequently experienced problem, reported for 35.3% and 39.5% of patients with EM or CM, respectively (*p* = 0.833) (Fig. 2a). Headache recurrence and MOH were the next most frequent problems with currently prescribed acute treatment in patients with CM; both were reported as a problem in significantly more patients with CM than with EM (*p* < 0.05). Lack of efficacy was also the most frequently identified problem with current preventive therapy among patients for whom PRF data on issues with preventive therapy were available (295 with EM; 21 with CM), being reported for 35.3% of patients with EM and 52.4% of those with CM (*p* = 0.131) (Fig. 2b). The next most frequent problems were drowsiness/sedation (reported as an issue in more patients with CM than with EM [p < 0.05]). A numerically higher proportion of patients with EM than with CM for whom data on issues with therapy were reported indicated that they had no predetermined issues with their currently prescribed acute (29.0% vs. 15.8%, respectively, *p* = 0.209) or preventive (26.8% vs. 23.8%; *p* = 0.529) treatment.

### Migraine burden

Among patients who provided data in PSC forms (477 with EM; 20 with CM), moderate-to-severe headache-related disability (MIDAS total score ≥ 11) was reported by significantly fewer individuals with EM than with CM (21.0% vs. 60.0%; *p* < 0.001). Mean MIDAS total score was lowest (4.8) in the EM 0–3 HDM subgroup but increased in the EM 4–7 HDM (11.0) and EM HDM 8–14 (15.7) subgroups, reaching 21.3 in the CM HDM ≥15 subgroup (higher scores signifying greater disability) (*p* < 0.001 for trend) (Table 4). Over 70% of patients with EM HDM 0–3 reported little or no disability (MIDAS total score 0–5), and only 4.7% reported severe disability (MIDAS total score > 20); in contrast, > 30% of patients in the EM 8–14 HDM subgroup and those with CM ≥15 HDM had severe disability (Table 4).

**Table 4:** Burden of illness in patients with episodic migraine and chronic migraine: patient self-completion form data

Patient-reported outcomes	Cohort				Total (*N* = 634)	*p*-value
	Episodic migraine 0–3 HDM (*n* = 331)	Episodic migraine 4–7 HDM (*n* = 183)	Episodic migraine 8–14 HDM (*n* = 86)	Chronic migraine ≥15 HDM (*n* = 34)		
MIDAS total score, mean (SD)	4.8 (12.6)	11.0 (26.2)	15.7 (18.3)	21.3 (22.0)	8.9 (19.4)	< 0.001^a^
MIDAS disability category, n (%)						< 0.001^b^
Little/no disability	191 (74.9)	86 (57.7)	29 (39.7)	6 (30.0)	312 (62.8)	
Mild disability	32 (12.5)	25 (16.8)	14 (19.2)	2 (10.0)	73 (14.7)	
Moderate disability	20 (7.8)	17 (11.4)	7 (9.6)	5 (25.0)	49 (9.9)	
Severe disability	12 (4.7)	21 (14.1)	23 (31.5)	7 (35.0)	63 (12.7)	
Derived Japanese EQ-5D utility score, mean (SD)	0.89 (0.16)	0.86 (0.18)	0.82 (0.19)	0.72 (0.23)	0.86 (0.18)	< 0.001^a^
EQ-5D overall VAS score, mean (SD)	73.8 (17.9)	72.5 (16.9)	68.8 (18.4)	60.8 (22.6)	72.1 (18.2)	< 0.001^a^
WPAI score, mean (SD)						
% Work time missed	4.5 (16.1)	4.4 (13.3)	2.8 (9.7)	8.5 (18.6)	4.4 (14.6)	0.562^a^
% Impairment at work	29.5 (25.5)	35.8 (27.6)	37.3 (29.4)	42.2 (25.1)	33.0 (26.9)	0.051^a^
% Overall work impairment	30.5 (26.7)	37.0 (28.9)	38.1 (29.4)	44.7 (28.0)	34.2 (28.0)	0.058^a^
% Activity impairment	32.4 (28.0)	40.1 (28.0)	46.1 (28.8)	55.0 (28.0)	37.7 (28.8)	< 0.001^a^

Continuous variables are reported as mean (SD) for non-missing observations; percentages are calculated as proportion of non-missing data. MIDAS score 0–5 = little or no disability; MIDAS score 6–10 = mild disability; MIDAS score 11–20 = moderate disability; MIDAS score > 21 = severe disability; higher EQ-5D utility and VAS scores indicate higher general health status and quality of life, respectively

EQ-5D, EuroQol-5 Dimensions; HDM, headache days per month; MIDAS, Migraine Disability Assessment; SD, standard deviation; VAS, visual analog scale; WPAI, Work Productivity and Activity Impairment

^a^Analysis of variance analyses

^b^Fisher’s exact test

EQ-5D utility and VAS scores were significantly (*p* ≤ 0.005) lower (indicating poorer general health status and HRQoL, respectively) in patients with CM than in those with EM. EQ-5D utility scores were highest in the EM 0–3 HDM subgroup (0.89), decreasing over the HDM 4–7 (0.86) and 8–14 subgroups (0.82), and lowest in patients with CM ≥15 HDM (0.72; *p* < 0.001 for trend) (Table 4). Similar findings were seen for HRQoL (EQ-5D VAS scores) (Table 4).

Patients with CM also experienced greater impairment and less productivity than those with EM, according to WPAI scores. Assessment of WPAI scores revealed that activity impairment increased with increasing numbers of HDM (from 0 to 3 HDM to ≥15 HDM; p < 0.001), whereas numerical increases were seen for impairment at work and overall work impairment. Work time missed showed no increase across the four HDM subgroups (Table 4).

## Discussion

The demographic profile of patients with EM and CM in Japan in this study differs in certain respects from that observed in large population-based studies in other countries [2, 4, 18, 19] and in another 2014 Adelphi Migraine DSP survey conducted in the USA [13].

The breakdown of patients classified as having EM (94.5%) and CM (5.5%) in the current study is consistent with the findings of the American Migraine Prevalence and Prevention (AMPP) study (a postal-based, longitudinal survey of respondents with headache) (94.5% and 5.5% [18]) and the International Burden of Migraine Study (IBMS) (an internet-based, cross-sectional multinational survey) (94.3% and 5.7% [4]); however, the US Adelphi Migraine DSP included a greater proportion of patients with CM (90.8% vs. 9.2% [13]).

In the current study, no significant difference in employment status was observed between patients with EM and those with CM. This contrasts with the AMPP study and the US Adelphi Migraine DSP survey, both of which found significantly lower levels of full-time employment in people with CM than in those with EM [13, 18]. Ford et al. also reported a significantly higher rate of unemployment among patients with CM than among those with EM.

Consistent with other studies [4, 13, 18, 19], women comprised three-quarters of the EM and CM cohorts in the current study. In Japan’s aging society, the expectation that women will contribute to society as workers is high. Findings in other studies [13, 18] that patients with CM are less likely to be employed (full or part time) than those with EM may suggest that severe migraine could pose an obstacle for an ambitious woman in Japan. However, migraine severity did not impact significantly on full-time employment status in the current study, which probably reflects the Japanese belief that working hard is a virtue (*gaman* in Japanese, which is generally translated as ‘perseverance’, ‘patience’, ‘tolerance’, or ‘self-denial’).

In common with the US Adelphi Migraine DSP [13], pain-related symptoms were the most frequent physician-reported migraine-related symptoms considered most troublesome to patients with EM or CM. However, apart from unilateral pain (seen in 51% and 38% of patients with EM and CM, respectively, in the Japanese Adelphi Migraine DSP and ~ 40% and ~ 43%, respectively, in the US Adelphi Migraine DSP), frequencies were notably lower in Japanese patients than in US patients. For example, pulsating/throbbing pain was reported in 21% and 38% of patients with EM and CM in the Japanese Adelphi Migraine DSP, compared with ~ 52% and ~ 53% of patients in the US Adelphi Migraine DSP [13]. In migraine-related clinical trials, patients are usually asked to identify their most bothersome symptom (photophobia, phonophobia, or nausea) in addition to pain. In the US Adelphi Migraine DSP, photophobia as a physician-reported migraine-related symptom considered most troublesome to patients was reported for 18% and 26% of patients with EM and CM, respectively; phonophobia was reported for 8% and 19%, respectively [13]; however, the prevalence of these symptoms was again much lower in patients in the Japanese Adelphi Migraine DSP (photophobia: 1.5% and 3.8%, respectively, and phonophobia: 0.9% and 0%, respectively). The concept of *gaman* may, again, have contributed towards these discrepant findings between patients with migraine in Japan and the USA, with concepts such as ‘tolerance’ and ‘self-denial’ resulting in Japanese patients being less likely to report migraine-related symptoms.

Comorbidity profiles varied between patients with EM and CM in Japan but, in contrast to other studies, not significantly. Anxiety and respiratory disorders were more common in patients with EM, and gastrointestinal problems and sleep disorders were more common in those with CM, as was depression, a known risk factor for CM [20]. In the AMPP study, people with CM were twice as likely as those with EM to be depressed (odds ratio [OR] 2.0; 95% confidence interval [CI] 1.67–2.40; *p* ≤ 0.001) or experience anxiety (OR 1.8; 95% CI 1.51–2.15; *p* ≤ 0.001). Similar findings were reported in the US Adelphi Migraine DSP [13]. The IBMS also reported higher rates of psychiatric disorders in patients with CM than in those with EM [4]. Respiratory disorders were reported to be more common in respondents with CM than in those with EM in the AMPP study [18].

Higher rates of cardiovascular risk factors (e.g., hypertension, high cholesterol, obesity) have also been reported in patients with CM than in those with EM [4, 13, 18], and obesity is known to be an important modifiable risk factor for CM [20]. Although rates in the current study did not differ between EM and CM cohorts, hypertension and hyperlipidemia were seen in notable percentages (~ 28% and ~ 20%, respectively) of patients with EM or CM with comorbidities, and ~ 10% of patients were obese. Correspondingly, 21–29% of patients were being prescribed antihypertensives/vasodilators, and 17–21% were receiving lipid-lowering agents for the treatment of comorbidities. Similar rates of hypertension and hyperlipidemia as comorbidities were also reported in patients with CM and EM in the US Adelphi Migraine DSP, but the rate of obesity was significantly higher in those with CM [13].

Although CM has been found in other studies to be associated with a greater societal economic burden than EM, to our knowledge, this is the first study to note that healthcare resource use did not differ notably between patients with EM or CM in Japan. In the US Adelphi Migraine DSP study, people with CM incurred significantly greater rates of physician visits, migraine-related hospitalizations, and surgical interventions than those with EM [13]. Physician visits in the previous 12 months were significantly higher for those with CM consulting with neurologists in the USA [13]. Similar findings were also reported in the AMPP and IBMS studies [4, 21].

Physician-reported prescriptions for acute treatment in patients with EM or CM, mainly comprising triptans and NSAIDs, were generally in line with JSN/JHS guidelines [11]. Loxoprofen was the most widely used NSAID for the acute treatment of patients with migraine; in contrast, ibuprofen and naproxen appeared to be more widely used for this purpose in the US Adelphi Migraine DSP survey [13]. In the current study, opioid use as acute therapy was low in both patients with EM and those with CM (0–0.2%), which again contrasts with the US Adelphi Migraine DSP survey findings (EM 8.3% vs. CM 13.9%; *p* < 0.001) [13]. However, both the Japanese and the US Adelphi Migraine DSP surveys found that patients with CM were significantly more likely to experience MOH than were those with EM. In the Japanese Adelphi Migraine DSP, MOH was reported by physicians to be a problem with acute treatment in significantly more patients with CM than with EM (*p* < 0.001).

Preventive therapy prescriptions were also generally in line with JSN/JHS guidelines [11]. It should be noted, however, that botulinum toxin and topiramate, which are used widely worldwide as preventive therapies for migraine (e.g., USA [13]), are approved in Japan for other diseases but not for migraine. Consequently, Japanese physicians have fewer treatment options for migraine prevention than physicians in other countries.

In the current study, nearly 60% and over 30% of patients with EM or CM, respectively, had never received a prescription for preventive therapy. While the percentage for patients with EM is similar to that reported in the US Adelphi Migraine DSP (50%), Japanese patients with CM were almost twice as likely never to have received a prescription for preventive therapy as the corresponding percentage reported in the US DSP (17%) [13]. Higher rates have been reported in population-based studies. For example, in the IBMS-II study, the percentage of patients with CM not currently using preventive therapy was 55% [19], and in the AMPP study, only 33.3% of patients with CM reported that they were current users of preventive therapy [22]. Although the percentages from population-based studies are based on patient self-reports, they nevertheless suggest that a large number of patients with CM are not receiving the long-term preventive therapy that patients with severe and/or frequent migraines require [23]. The findings in the current study that over half of the patients with CM who were not receiving preventive therapy were consulting an internist rather than a neurologist could possibly indicate that non-specialist physicians or patients were reluctant to initiate such therapy. For example, patients and/or physicians may have concerns over the potential for adverse events with such long-term treatment [24, 25]. These findings may therefore highlight a need for ways of increasing the willingness of patients to accept and for physicians to initiate pharmacological migraine-preventive therapy.

According to physician-reported data, notable proportions of patients with EM or CM experienced at least one problem with their current acute (> 50%) or preventive (> 20%) therapy. Lack of efficacy was the mostly frequently identified problem with both acute and preventive treatment, occurring in over one-third of patients. This finding that many patients with migraine experienced problems with their therapy could be reflected in the number of prescriptions for second-, third-, or even fourth- (or higher)-line acute or preventive therapy observed in patients with EM or CM (although it should be borne in mind that the number of patients who received more than one line of therapy could have been influenced by the inclusion of a 10% oversampling of those who had failed at least one prior preventive treatment). In the previously mentioned AMPP study, > 40% of people with EM were found to have at least one unmet need with their current acute treatment, which included satisfaction with therapy (assessed as lack of efficacy, tolerance, or overall satisfaction with the medication); people with one unmet need (OR 1.49, 95% CI 1.22–1.82) or two or more unmet needs (OR 2.21, 95% CI 1.77–2.76) were more likely than those with no unmet needs to have used triptans in the past 3 months [26]. Lack of efficacy, tolerability, or both have also been reported to result in poor adherence and persistence with preventive medication in people with EM or CM [19, 23, 27]. In the current study, significantly more patients with CM than with EM had switched or discontinued acute or preventive treatment, and twice as many of those with CM than with EM were receiving current prescriptions for both acute and preventive treatments, findings that possibly reflect higher levels of unmet treatment need in people with CM. However, it is possible that the inclusion of a 10% oversampling of those who had failed at least one prior line of preventive treatment may have influenced this finding since the CM group was over-represented by these patients (22% vs. 10% of the EM group).

Headaches occurring in people with migraine have a substantial negative effect on the general health, HRQoL, and functioning of those affected [3, 4]. Our findings are in agreement with those of the US Adelphi Migraine DSP [13] and reveal a general trend for increased impairments in the patient-reported outcomes of headache-related disability, activity impairment, work productivity, and function as the number of HDM increased, with levels in the EM 8–14 HDM subgroup approaching or equaling those in the CM ≥15 HDM subgroup. For example, patients in the both EM 4–7 HDM and EM 8–14 HDM subgroups, as well as those in the CM ≥15 HDM subgroup, were impaired at work for ~ 30% of the time. In contrast, the lack of a trend for an increase in WPAI scores for work time missed across HDM subgroups could again attest to the importance of *gaman* in Japanese culture. Such a finding could impact economic assessments of migraine on work productivity in Japan.

Impairments in general health status and HRQoL also increased with the frequency of HDM. Other studies [13, 28] have reported similar findings.

### Strengths/limitations

One strength of this study has been the use of real-world data collected using a standardized methodology (as part of the Adelphi Migraine DSP), thus allowing direct comparison of migraine treatment patterns and disease burdens between Japan and other countries. Adelphi DSPs are large, multinational, cross-sectional observational studies of clinical practice providing valuable data on a range of common chronic diseases to supplement the findings of larger population-based studies [12]. As both clinical and patient-reported data are collected, we consider the Adelphi Migraine DSP to be an appropriate database for answering the research questions posed in this study. DSP data have previously helped elucidate treatment patterns in people with migraine in the USA, Germany, France, and Japan [13, 29–33].

All patient participants in the current study had been diagnosed with migraine by a physician; hence, the study findings should be considered generalizable to the consulting population of patients with EM or CM in Japan. However, generalizability is limited by the fact that study sites were selected based on the volume of patients with migraine routinely seen and, hence, consulting physicians were experienced in treating migraine.

Other limitations of the study include that the data are cross-sectional in nature; however, PRF data were considered suitable for the assessment of treatment patterns given that they were supplied by the physician and patient records could be referred to for patient history. Additionally, the Japanese Adelphi Migraine DSP was conducted over a 4-month period and included a limited number of physician and patient participants, especially patients with CM. This may account for the lack of many significant differences between the EM and CM study populations that have been identified in population-based studies involving larger migraine populations (e.g., the AMPP and IBMS studies). There is also a potential for selection bias associated with the oversampling of patient participants who had failed at least one prior line of preventive therapy.

## Conclusions

Managing migraine is challenging for both patients and physicians. As a guide for healthcare professionals, this analysis of the Japanese Adelphi Migraine DSP data provides a real-world snapshot of information on the clinical characteristics, disease burden, and treatment patterns of people with EM and CM being treated in clinical practice in Japan in 2014. In Japan, preventive treatment patterns differ in people with EM compared with those with CM, and both EM and CM pose notable disease burdens. Our data suggest that many patients were not receiving appropriate preventive therapy. These findings, together with the complexity of migraine, the heavy economic burden the disease places on healthcare resources, and the impact on people’s ability to function, suggest an unmet need for more effective migraine therapies to reduce the burden of this disease. Importantly, further studies/research are needed to explore factors affecting patients’ willingness to take preventive treatments and improve long-term outcomes.

## Additional files

Additional file 1: **Table S1.** Drug classes being taken for the treatment of comorbidities by ≥5% of patients with episodic and chronic migraine and any comorbidity: physician-reported data. (DOCX 15 kb)

Additional file 2: **Figure S1.** Comorbid medical conditions reported in > 5% of patients with episodic or chronic migraine with any comorbid medical condition: physician-reported data provided for all patients with comorbidities (EM *N* = 455; CM *N* = 29). GI, gastrointestinal; COPD, chronic obstructive pulmonary disease. (TIF 1921 kb)

Additional file 3: **Figure S2.** Number of acute and prophylactic treatment lines ever received in patients with episodic migraine (EM) or chronic migraine (CM): physician-reported data. Lines of treatment could have included different classes of medication for migraine. ^a^Fisher’s exact test. (TIF 1956 kb)

## Abbreviations

AMPP: American migraine prevalence and prevention
ANOVA: Analysis of variance
CI: Confidence interval
CM: Chronic migraine
DSP: Disease-Specific Programme
EM: Episodic migraine
EQ-5D: EuroQol-5 dimensions
HDM: Headache days per month
HRQoL: Health-related quality of life
IBMS: International burden of migraine study
IHS: International headache society
JHS: Japanese headache society
JSN: Japanese society of neurology
MIDAS: Migraine disability assessment
MOH: Medication-overuse headache
NSAID: Nonsteroidal anti-inflammatory drug
OR: Odds ratio
PRF: Patient record form
PSC: Patient self-completion
SD: Standard deviation
VAS: Visual analog scale
WPAI: Work Productivity and Activity Impairment
YLD: years lived with disability
