# Supplementary material for: Real-world treatment patterns and patient-reported outcomes in episodic and chronic migraine in Japan: analysis of data from the Adelphi migraine disease specific programme
Source: J Headache Pain. 2019 Jun 7;20(1):68. doi: 10.1186/s10194-019-1012-1 (PMC6734304; doi:10.1186/s10194-019-1012-1)
Supplement: Supplementary file 1 — Table S1. Drug classes being taken for the treatment of comorbidities by ≥5% of patients with episodic and chronic migraine and any comorbidity: physician-reported data. (DOCX 15 kb) [file 10194_2019_1012_MOESM1_ESM.docx]

**Additional file 1: Table S1.** Drug classes being taken for the treatment of comorbidities by ≥5% of patients with episodic and chronic migraine and any comorbidity: physician-reported data

|  | Cohort | |  |  |
| --- | --- | --- | --- | --- |
| Medications | Episodic migraine (N=455) | Chronic migraine (N=29) | Total (N=484) | p-value |
| SSRI/SNRI antidepressants | 55 (12.5) | 2 (7.1) | 57 (12.2) | 0.558^a^ |
| Sedatives | 51 (11.6) | 3 (10.7) | 54 (11.6) | 1.000^a^ |
| Analgesics | 8 (1.8) | 3 (10.7) | 11 (2.4) | **0.023^a^** |
| Antihistamines | 33 (7.5) | 1 (3.6) | 34 (7.3) | 0.711^a^ |
| Antihypertensives/  vasodilators | 93 (21.2) | 8 (28.6) | 101 (21.6) | 0.357^b^ |
| Calcium antagonists | 30 (6.8) | 2 (7.1) | 32 (6.9) | 1.000^a^ |
| Oral hypoglycemics | 23 (5.2) | 1 (3.6) | 24 (5.1) | 1.000^a^ |
| Lipid-lowering agents | 73 (16.6) | 6 (21.4) | 79 (16.9) | 0.447^a^ |
| Respiratory/asthma therapies | 25 (5.7) | 1 (3.6) | 26 (5.6) | 1.000^a^ |
| Antacids/H_2_ receptor antagonists | 41 (9.3) | 4 (14.3) | 45 (9.6) | 0.332^a^ |
| Proton pump inhibitors | 32 (7.3) | 3 (10.7) | 35 (7.5) | 0.456^a^ |

Data are n (%); percentages are calculated as proportion of non-missing data (episodic migraine, N=439; chronic migraine, N=28; total, N=467)

SNRI, serotonin-norepinephrine reuptake inhibitor; SSRI, selective serotonin reuptake inhibitor

^a^Fisher’s exact test

^b^Chi-squared test
