# Supplementary figures and images for: Real-world treatment patterns and patient-reported outcomes in episodic and chronic migraine in Japan: analysis of data from the Adelphi migraine disease specific programme
Source: J Headache Pain. 2019 Jun 7;20(1):68. doi: 10.1186/s10194-019-1012-1 (PMC6734304; doi:10.1186/s10194-019-1012-1)

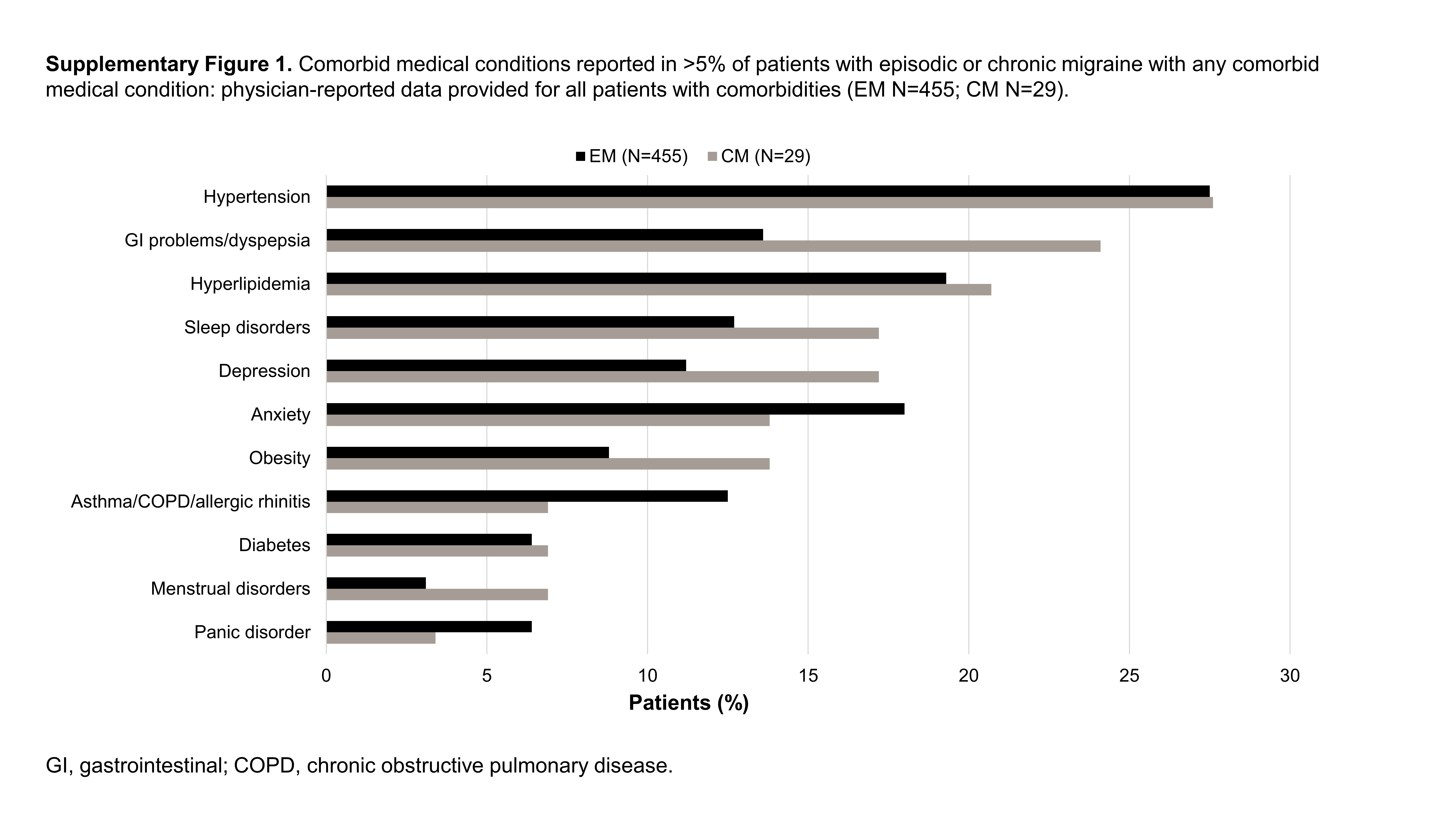

Supplement: Supplementary file 2 — Figure S1. Comorbid medical conditions reported in > 5% of patients with episodic or chronic migraine with any comorbid medical condition: physician-reported data provided for all patients with comorbidities (EM N = 455; CM N = 29). GI, gastrointestinal; COPD, chronic obstructive pulmonary disease. (TIF 1921 kb) [file 10194_2019_1012_MOESM2_ESM.tif]

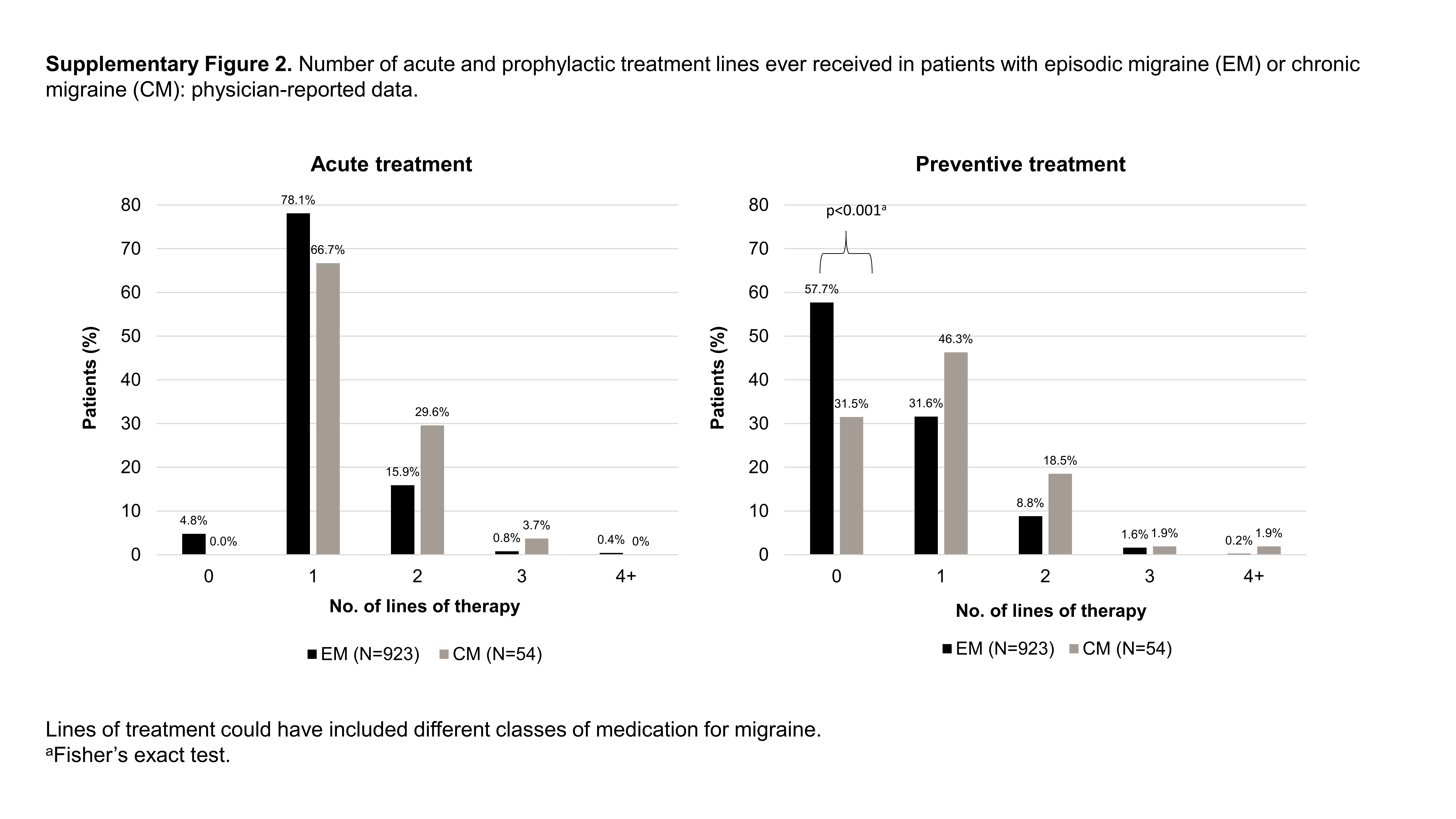

Supplement: Supplementary file 3 — Figure S2. Number of acute and prophylactic treatment lines ever received in patients with episodic migraine (EM) or chronic migraine (CM): physician-reported data. Lines of treatment could have included different classes of medication for migraine. aFisher’s exact test. (TIF 1956 kb) [file 10194_2019_1012_MOESM3_ESM.tif]
